# Prioritizing information topics for relatives of critically ill patients: Cross-sectional survey among intensive care unit relatives and professionals

**DOI:** 10.1007/s00508-018-1377-1

**Published:** 2018-08-09

**Authors:** Magdalena Hoffmann, Anna K. Holl, Harald Burgsteiner, Philipp Eller, Thomas R. Pieber, Karin Amrein

**Affiliations:** 1 Division of Endocrinology and Diabetology, Department of Internal Medicine, Medical University of Graz, Graz, Austria; 2 Executive Department for Quality and Risk Management, University Hospital Graz, Graz, Austria; 3 Research Unit for Safety in Health, Medical University of Graz, Graz, Austria; 4 Department for Psychiatry, University Hospital Graz, Graz, Austria; 5 Institute for Digital Competence and Media Education, University College of Teacher Education Styria, Graz, Austria; 6 Intensive Care Unit, Department of Internal Medicine, Medical University of Graz, Graz, Austria; 7 Joanneum Research, Graz, Austria

**Keywords:** Intensive care unit, Relatives, Information, Communication, Health literacy

## Abstract

A patient’s admission to an intensive care unit (ICU) has a significant impact on family members and other relatives. In order for them to be able to cope with such a stressful situation, the availability of appropriate understandable and accessible information is crucial. The information asymmetry between relatives and medical professionals may adversely affect satisfaction of relatives and their risk of subsequent anxiety, depression and stress symptoms. The aim of this study was therefore to understand which topics are most important to the relatives of ICU patients and to quantify the perceptions of medical professionals regarding the information needs of relatives. A cross-sectional survey was conducted in 2015. The survey had 42 questions, such as ‘diagnosis’, ‘treatment’, ‘comfort’, ‘family’ and ‘end of life’. In total, the survey was handed out to four different groups. A total of 336 persons answered the survey (26 relatives, 28 ICU physicians, 202 ICU nurses and 80 ICU medical professionals in a closed Facebook© group [Facebook, Menlo Park, California, USA]). Relatives ranked the five most important topics as follows: ‘recent events (crisis)’, ‘my participation’, ‘contamination in hospital’, ‘physical pain’, and ‘probability’. Several significant differences (*p*<0.001) were detected, for example for the topics fever, medication, recent events (crisis), appointments, relapse, and investigations. Even the topic with the lowest ranking (religion) had a score of 3.15 (min. 1.00, max. 5.00) among relatives. The ICU professionals appear to have divergent opinions regarding the most important topics for ICU relatives as compared to relatives themselves.

**Electronic supplementary material:**

The online version of this article (10.1007/s00508-018-1377-1) contains supplementary material, which is available to authorized users.

## Introduction

Relatives in intensive care units (ICU) are important partners in the decision-making underlying the treatment of critically ill patients. They can be a significant resource by providing information, and in the care and rehabilitation of patients [1, 2], but the critical illness of a close relative negatively affects them too [3]. Many ICU patients have few or no memories of their ICU stay, but their relatives often experience sleep problems, anxiety and feelings of helplessness. In a recent study, relatives suggested that ‘more information’ [4] would be helpful to improve their poor sleep quality.

Currently, there often is a substantial information asymmetry between health professionals and relatives/patients. The health literacy of the relatives is undoubtedly an important co-factor in the communication process [5, 6], as 50% of relatives fail to understand healthcare staff communication [7]. This could have an impact on relatives as well as on the rehabilitation of the patients [8] and may lead to symptoms of anxiety, stress, depression, and sleep problems [4, 9–12]. Relatives often develop family intensive care unit syndrome (FICUS) [13], defined as maladaptive reasoning, high-intensity emotions, sleep deprivation, personal and family conflicts, cognitive bias and anticipatory grief [13]. Most family members report some levels of anxiety, depression and stress [11, 14]. Importantly, an association between a perceived lack of information and symptoms of post-traumatic stress disorder (PTSD) has been reported [4, 15]. It is therefore important to provide appropriate and sufficient information to families of ICU patients [16].

A recent Italian study demonstrated that structured information (verbally, in writing and through online media) could reduce stress and post-traumatic stress [17]. Al-Mutair et al. concluded in a literature review that family members ranked the need for information as the most important need second only to insurance [18]; however, detailed information on the subjective importance of different topics is largely absent in the literature, especially in German speaking countries. It was hypothesized that variations in subjective importance regarding different topics exist. Furthermore, differences between relatives and medical professionals were expected in their respective evaluation of the importance of topics. This survey aimed to clarify which topics are subjectively most important to relatives of critically ill patients and to compare the subjective perceptions of relatives with those of ICU professionals.

## Materials and methods

The study was approved by the Medical University of Graz ethics committee (EK 27-317 ex 14/15).

### The survey

The survey was based on the survey by Peigne et al. 2011 [19], conducted in 14 ICUs across France, which aimed to identify important questions asked by family members of critically ill patients. In this survey. 9 topics and 42 subtopics were included. The survey in this study included 42 questions on specific topics and assessed sociodemographic baseline information. A short explanation was included alongside each question for better understanding. For instance, the topic ‘my participation’ was explained as ‘what can I do to help my relative,’ while the topic ‘crisis’ was described as ‘deterioration of vital signs or psychological symptoms’. We used a 5-point scale from 1 not important, to 5 very important and with the option of ‘not relevant’. After a review process with medical professionals, the survey was pretested with 10 individuals without any medical background. A detailed description of the survey is presented in Table 1, and the original version of the survey can be found in the electronic supplementary material. The survey was distributed in summer 2015 over a period of 3 months and the aim was to include as many respondents as possible in this time frame.

**Table 1:** Detailed description of the survey categories

No. of section	Name of the section	Description
1	Sociodemographic data	The questions referred to the sex, age, relationship to the critically ill patient, previous experience with the ICU, living together in the household, education^**a**^
2	Diagnosis	Neurological status, fever, diseases, appearance, vital signs, examinations^**b**^
3	Treatment and Therapy	Operations, treatment and therapy, weaning off the respirator, respirator, medication^**b**^
4	Prognosis	Duration of illness, death and grief, probabilities and assumptions, rights to information and information, crises^**b**^
5	Comfort	Decrease mental stress, well-being, physical pain, nutrition, sleep^**b**^
6	Interaction	Speaking, responding, touching, listening, my participation^**b**^
7	Communication	Dates in the intensive care unit, information received, news, team, telephone^**b**^
8	Family/relatives	Visiting times, contamination, family conference, stress and worry, religion^**b**^
9	Post-ICU	Length of stay, relapse, sequelae, relocation, reminders^b^
10	End of life	Futility, death and grief^b^
12	Internet use	Use of the internet to learn about health issues^a^
13	Open space for extra questions	Free space for open/new questions, topic forgotten, a message^c^

*ICU* intensive care unit

^a^Closed question, multiple choice question

^b^5-point scale, not important to very important, with the option of ‘not relevant’

^c^Free space

### The population

In total, four groups answered the survey: relatives of ICU patients, medical professionals including ICU physicians, ICU nurses at the Medical University Graz, Austria and a Facebook© group with a focus on intensive care professionals.

### Relatives

In this study three ICUs at the University Hospital of Graz (a large tertiary care facility in Styria, the south-eastern region of Austria) participated. The study included a general ICU with 11 beds, a cardiac ICU with 9 beds and a neurology ICU with 8 beds. To be included, relatives of critically ill patients had to be aged between 18 and 80 years and living in Styria. Only the relatives of patients who were predicted by the attending physician to stay in the ICU for at least 72 h were included. Exclusion criteria were a lack of proficient German and a do not resuscitate order on the patient. The paper-based survey was handed out to the relatives by the treating consultant at their second or third meeting. A neutral envelope was given to the relatives to allow them to return the survey anonymously after completion.

### Medical professionals

The three medical professional groups completed the survey online using the free version of the platform “SurveyMonkey” (www.surveymonkey.de). The ICU physicians received an email invitation with a link and a request to send the email to other ICU physicians. All ICU nurses from the Medical University of Graz received an email invitation with a link. The third group of medical professionals were members of two ICU-related Facebook© groups called *Intensivpflege und Anästhesiepflege—Community & Forum 05/2012* and, *Intensivpflege—24/7*. Both groups are closed membership, i. e. require an approved membership request to join. The invitation to complete the survey alongside with the link was posted into these two groups.

### Statistical analysis

Survey data were analyzed using descriptive statistics for the total cohort and for each of the four groups. Descriptive data analysis was performed with Microsoft Excel 2013 (Microsoft Corporation, Redmond, WA, USA). A Mann-Whitney U‑test was performed with SPSS (IBM SPSS Statistics 24© IBM Corp. 1989, 2016, Armonk, NY, USA). An alpha level adjustment for multiple comparisons according to C.E. Bonferroni was done. For significant differences the *p* value must be <0.016.

## Results

In total, 336 persons participated. The survey was answered by 26 relatives (response rate 50%), 28 physicians (response rate not calculable) 202 nurses (response rate 52%) and 80 ICU professionals at the Facebook© group (response rate not calculable). For each question 80% (minimum) participation was reached. A detailed description of participating relatives is presented in Table 2. The participating medical professionals are described in Table 3.

**Table 2:** Baseline characteristics of the relatives of ICU patients

Domain	Relatives (*N* = 26)	%
Gender	Female	61.5
	Male	38.5
Country of origin	Austria	100
Age (years)	18–40	23.0
	41–60	50.0
	61–80	19.0
	Unknown	8.0^**a**^
Relationship to the patient^a^	Wife/husband	38.5
	Sister/brother	4.0
	Parents	19.0
	Son/daughter	15.0
	Other	19.5
	Unknown	4.0^**a**^
ICU experience	Yes	54.0
	No	35.0
	Unknown	8.0^**a**^
Living in the same household	Yes	50.0
	No	42.0
	Unknown	8.0^**a**^
Level of education	Primary school/compulsory school	11.5
	Graduated secondary school/apprenticeship	61.5
	Apprenticeship with management qualification/college/university	19.0
	Unknown	8.0^**a**^
Internet use for information about current ICU stay of a relative	Yes	38.0
	No	54.0
	Unknown	8.0^**a**^

Data are presented in %

^a^Some data are missing for 2 relatives

**Table 3:** Baseline characteristics of the ICU medical professionals

Domain	Nurses (*N* = 202)	Physicians (*N* = 28)	Facebook© group (*N* = 80)
*Gender (in %)*			
Female	78.7	36.0	72.5
Male	21.3	64.0	27.5
*Age in years*			
Mean	34.8	41.2	35.6
*Work experience in years*			
Mean	12.7	13.8	11.8
*Country of origin in percentage terms (in %)*			
Austria	100	96.4	10.0
Germany	0	3.6	88.7
Switzerland	0	0	1.3

Medical professionals. Data are presented in % terms or years, as relevant

For relatives, the five most important topics (ranked by mean) were ‘recent events (crisis)’ (e. g. acute deterioration of physical indicators, such as fever or blood pressure), ‘my participation’ (e. g. what can I do to help), ‘contamination in the hospital’ (e. g. what is important for me to know about hand hygiene or isolation), ‘physical pain’ (e. g. does the patient have pain and what will be done to prevent/treat pain) and ‘what happens next’ (e. g. discharge from ICU).

The topics with the lowest ranking were ‘religion’ (e. g. religious support), ‘memory’ (e. g. diary keeping at ICU) and ‘ICU news’ (e. g. news about the specific ICU). A detailed description of the relatives’ ratings is presented in Table 4.

**Table 4:** Results of ratings given by relatives to each of the 42 questions

Ranking	Topic	Mean	Ranking	Topic	Mean
1	Crisis	4.90	22	Disease	4.43
2	My participation	4.84	23	Futility	4.42
3	Contamination	4.71	24	Talking	4.40
4	Physical pain	4.70	25	What treatment?	4.39
5	Probability	4.67	26	Weaning	4.38
6	Appointments	4.65	27	Length of stay	4.38
7	Relapse	4.65	28	Investigations	4.35
8	Touching	4.64	29	Fever	4.32
9	Answering	4.62	30	Food	4.25
10	Telephone	4.62	31	Supplying comfort items	4.19
11	Transfer	4.62	32	Death	4.15
12	Hearing	4.60	33	Appearance	4.14
13	Medication	4.59	34	Tubes and machines	4.09
14	Recovery	4.57	35	Decision-making	4.05
15	Visits	4.55	36	Information and rights to information	4.00
16	Vital signs	4.50	37	Sleep	4.00
17	Staff members	4.50	38	Relatives’ distress	3.80
18	Sequelae	4.50	39	Being informed	3.76
19	Neurologic status	4.48	40	News	3.67
20	Psychological distress	4.48	41	Memory	3.37
21	Surgery	4.47	42	Religion	3.15

Important information topic ranked by relatives. Participants rated each question from ‘not important at all’ (1) to ‘very important’ (5) with the option of ‘not relevant’. Data presented as mean

The ICU physicians considered the five most important topics for relatives to be ‘telephone’ (e. g. where and when can I call), ‘neurological status’ (e. g. consciousness, visual capacity), ‘hearing’ (e. g. can my relative hear me), ‘futility’ (e. g. death and grief) and ‘visiting’ (e. g. who can visit at which times). The ICU nurses rated ‘visiting’, ‘telephone’, ‘hearing/neurological status’ (equal rates), ‘touching’ (can I touch my relative)/‘physical pain’ (equal rates) as the five most important topics. For the professionals of the Facebook© group, the top 5 were ‘touching’, ‘hearing’, ‘neurological status’, ‘futility’ and ‘visiting’.

The overlap of topics between ICU professionals and relatives was limited. None of the top 5 topics of the relatives featured in the top 5 of medical professionals. The topic ‘recent events (crisis)’ is considered the most important topic by relatives, for instance, with a mean value of 4.90; however, it did not feature among medical professionals as an important topic, with mean rankings of 3.85 (physicians), 3.95 (nurses), and 4.14 (Facebook© group). This corresponds to respective positions in rankings of 25, 22, and 23 out of 42. A detailed description of the results is presented in Table 5 and 6.

**Table 5:** Top 5 ratings given by relatives compare with physicians, nurses and Facebook group

Domain	Subdomain	Relatives		Physicians		Nurses		Facebook© group	
		Mean	Ranking	Ranking	*P* value	Ranking	*P* value	Ranking	*P* value
Prognosis	Recent events (crisis)	4.90	1	25	<0.001^a^	22	<0.001^a^	23	<0.001^a^
Prognosis	My participation	4.83	2	11	0.074	16	<0.001^a^	11	0.020
Family	Contamination	4.70	3	18	0.003^a^	21	0.001^a^	8	0.019
Comfort	Physical pain	4.68	4	11	0.326	6	0.305	6	0.246
Prognosis	Probability	4.65	5	15	0.093	19	0.001^a^	20	0.027

Data are ranked by mean values

^a^Significant differences *p-*value <0.016

**Table 6:** All results: ratings by relatives, physicians, nurses and Facebook©group

Subdomain	Relatives (*N* = 26)		Physicians (*N* = 28)			Nurses (*N* = 202)			Facebook©group (*N* = 80)		
	MeanN	±SD	MeanN	±SD	*P* value	Mean	±SD	*P* value	Mean	±SD	*P* value
Neurologic status	4.48	0.99	4.71	0.46	0.725	4.60	0.74	0.737	4.68	0.60	0.555
Fever	4.32	1.17	2.82	0.90	<0.001*	3.43	0.94	<0.001*	3.15	0.96	<0.001*
Disease	4.43	1.12	4.18	0.77	0.052	4.13	0.83	0.017	4.26	0.86	0.107
Appearance	4.14	1.15	3.61	0.96	0.043	3.79	0.92	0.047	3.80	0.89	0.054
Vital signs	4.50	1.14	3.93	0.86	0.003*	4.14	0.95	0.015*	4.04	0.79	0.002*
Investigations	4.35	1.03	3.79	0.83	0.011*	3.51	1.02	<.001*	3.43	0.93	<.001*
Surgery	4.47	1.07	4.44	0.70	0.324	4.32	0.83	0.111	4.51	0.65	0.436
What treatment?	4.39	1.03	3.89	0.97	0.032	3.66	1.07	0.001*	3.88	0.93	0.007*
Weaning	4.38	1.07	4.19	0.74	0.111	4.01	0.98	0.037	4.42	0.71	0.567
Tubes and machines	4.09	1.34	3.07	1.07	0.003*	3.35	1.17	0.002*	3.45	1.14	0.012*
Medication	4.59	1.01	3.22	1.01	<0.001*	3.09	1.00	<0.001*	3.38	1.00	<0.001*
Recovery	4.57	0.66	4.52	0.58	0.717	4.41	0.75	0.308	4.37	0.78	0.295
Death	4.15	1.31	4.59	0.69	0.538	4.39	0.87	0.876	4.49	0.87	0.519
Probability	4.67	0.58	4.33	0.73	0.093	4.02	0.89	0.001*	4.26	0.80	0.027
Information and rights to information	4.00	1.18	3.37	0.97	0.036	3.87	1.13	0.537	3.82	1.16	0.373
Recent events	4.90	0.30	3.85	0.91	<0.001*	3.95	0.93	<0.001*	4.14	0.86	<0.001*
Psychological distress	4.48	0.81	4.11	0.89	0.161	4.14	0.76	0.035	4.41	0.60	0.376
Supplying comfort items	4.19	1.21	3.96	1.02	0.366	4.03	0.89	0.155	4.31	0.80	0.778
Physical pain	4.70	0.66	4.52	0.85	0.326	4.59	0.65	0.305	4.57	0.63	0.246
Food	4.25	0.91	3.37	0.97	0.004*	3.60	0.97	0.007*	3.79	0.87	0.049
Sleep	4.00	1.26	3.62	0.90	0.089	4.08	0.87	0.725	4.28	0.79	0.653
Talking	4.40	1.06	4.50	0.51	0.470	4.24	0.83	0.198	4.39	0.70	0.447
Answering	4.62	0.86	4.50	0.59	0.082	4.33	0.79	0.030	4.42	0.77	0.088
Touching	4.64	0.85	4.63	0.74	0.815	4.59	0.71	0.386	4.83	0.41	0.590
Hearing	4.60	0.88	4.69	0.55	0.738	4.60	0.65	0.476	4.76	0.47	0.966
My participation	4.84	0.50	4.48	0.80	0.074	4.10	0.94	<0.001*	4.47	0.73	0.020
Appointment	4.65	0.70	3.73	0.83	<0.001*	3.71	1.05	<0.001*	3.90	0.98	0.003*
Being informed	3.76	1.26	3.48	0.80	0.386	3.57	1.14	0.391	3.45	0.99	0.181
News	3.67	1.39	2.32	0.90	0.001*	2.58	1.17	<0.001*	2.46	0.93	<0.001*
Staff members	4.50	0.80	3.59	0.97	0.002*	3.09	1.11	<0.001*	3.21	1.00	<0.001*
Telephone	4.62	0.74	4.78	0.51	0.408	4.65	0.67	0.949	4.56	0.64	0.399
Visits	4.55	0.74	4.65	0.56	0.940	4.67	0.58	0.544	4.62	0.68	0.734
Contamination	4.71	0.72	4.11	0.80	0.003*	3.98	1.11	0.001*	4.52	0.66	0.119
Decision-making	4.05	1.08	4.41	0.69	0.316	3.84	1.09	0.357	4.30	0.75	0.612
Relatives’ distress	3.80	1.20	4.19	0.74	0.302	3.88	0.99	0.922	4.28	0.76	0.149
Religion	3.15	1.35	3.15	0.92	0.836	3.32	1.04	0.677	3.47	0.98	0.408
Length of stay	4.38	0.86	4.07	1.00	0.256	3.89	0.89	0.015*	3.66	0.96	0.002*
Relapse	4.65	0.67	3.48	1.00	<0.001*	3.66	1.10	<0.001*	3.91	0.89	<0.001*
Sequelae	4.50	0.83	4.07	0.68	0.033	3.49	1.01	<0.001*	3.91	0.90	0.006*
Transfer	4.62	0.67	3.85	0.83	0.001*	3.59	1.07	<0.001*	4.00	0.87	0.003*
Memory	3.37	1.64	2.81	0.96	0.200	2.64	1.13	0.034	3.21	0.99	0.432
Futility	4.42	0.96	4.67	0.68	0.172	4.54	0.70	0.396	4.67	0.62	0.185

Between relatives and the other groups a Mann-Whitney U‑test was performed

*Significant differences *p-*value <0.016.

Missing data: all available data were included in the statistical analysis.

Minimum response rate for each question was >80% in this survey

Across all questions, the relatives generally assigned a higher importance to the topics than the medical professionals, with an average (mean) grade of 4.35 on the importance scale, compared to physicians (3.94), nurses (3.90), and the Facebook© group (4.05). Highly significant differences (all *P* < 0.016) were detected between relatives and physicians, e. g. in the domains fever, medication, recent events (crisis), appointment and relapse. Significant differences were also detected between relatives and nurses in the domains fever, investigations, medication, recent events, my participation, appointment, news, staff members, relapse, sequelae and transfer. The highest significant differences between relatives and the Facebook© group were fever, investigations, medication, recent events, news, staff members, relapse.

## Discussion

In this survey at a large Austrian tertiary care hospital, it was found that the specific topics that relatives prioritize as most relevant diverge greatly from those prioritized by medical professionals. Furthermore, the fact that relatives consistently ranked information topics as more important than professionals indicates that information needs may be higher than perceived by ICU professionals. In this study, the medical professionals in the Facebook© group achieved a greater match with information needs of the relatives than the other staff surveyed. This phenomenon cannot be explained within the present investigation. A hypothesis could be that the Facebook© group exchanges information on social media about intensive care topics voluntarily and more intensively than others and therefore may be better informed about the needs of relatives.

### Implications for structuring communication between relatives and medical professionals

Medical professionals should strive for adequate, easy to understand communication and information sharing with affected relatives [17]; however, communicating with relatives of critically ill patients often presents challenges due to time constraints, high emotional demands on both sides and variable levels of health literacy. While the information needs of relatives are undoubtedly high, they should also not be overburdened with irrelevant or overly complex information.

The results of this study provide valuable hints as to which information topics matter most to relatives [20]. Familiarity with ICU relatives’ subjective rankings of information topics may help to address their needs effectively and prioritize topics at a very critical time [31]; however, high-quality dialogue with relatives requires more than simply addressing the right topics. Beyond the content level (subject matter), appropriate linguistic-interactive level (conversation), a psychosocial level (relationship) and a suitable framing of conversation (environment) are also necessary [21, 22]. The choice of words matters, e. g. a hypertensive episode may be understood to mean a severe threat or crisis event by relatives, while being trivial to healthcare workers. Furthermore, unless relatives understand the available information, they will not be able to pass it on to others or participate in treatment decisions [23]. Little is known about the consequences of deficiencies at these communication levels or the link between poor information provision on the part of medical professionals and unsupportive interactions with families [24]. Nonetheless, a study by Curtis et al. showed that communication training for ICU professionals could improve relatives’ satisfaction and reduce symptoms of stress, anxiety and depression [25].

A further challenging aspect is that the provision of adequate information is time-consuming for medical professionals [7, 26, 27]. On the other hand, the increasing availability of high-quality online resources and widespread use of smartphones has the potential to reduce the burden on professionals. A 2014 study found that 50% of ICU patients’ relatives had used the internet for information purposes within the first days of ICU treatment [28]. This indicates that relatives already use the internet as an important source of information.

The findings of this study also helped in constructing a website to offer continuously available information for all levels of health literacy. This website will be tested in a multicenter and international randomized controlled trial (www.clinicaltrials.gov, NCT02931851). The findings may also support further research on improving access to information for relatives [29–32]. In this respect, information asymmetries could be reduced and help relatives to become better informed partners in decision making [7]. Furthermore, a reduction in anxiety, stress, depression and sleep problems in relatives may be achievable [4, 17, 33].

The limitations of the present study are the relatively low number of participating relatives and physicians, and the fact that the survey was limited to fluent German speakers and restricted to German speaking countries. Another important limitation of the study is the variations in response rates of participant groups. In particular, the low response rate among physicians indicates the possibility that those with a higher sensitivity to the issues addressed here were self-selected into the survey. Also, while levels of health literacy in the geographical region of the study are below the European and Austrian average [5], an individual assessment of participants’ competencies was not conducted. In the data analysis, all answered questions were included in the evaluation. Each question was answered by at least >80% of the participants. Another important limitation is that the majority of Facebook© group members come from Germany. Austria is a secular, yet predominantly Christian country but in our survey the topic religion was ranked very low. This could be a bias due to the low number of participants. Another possible bias is the preselection of relatives (e.g. no relatives of do not resuscitate patients). The chosen inclusion criteria were based on criteria for the future study ICU Families—RCT (ClinicalTrials.gov Identifier: NCT02931851).

## Conclusion

In this study, a broad variety of topics was subjectively relevant to ICU relatives. There was a substantial discrepancy between relatives and ICU professionals in the subjective importance of topics: not a single top five topic for relatives featured among the top five topics for medical professionals. In the clinical routine it may be useful to focus conversations on the most relevant topics. When subjectively low-rated topics are objectively important (and vice versa), the recognition of this misconception should be openly discussed with family members and this may help reduce unrealistic expectations. Future larger studies should evaluate the information needs of ICU relatives in different regions, ethnicities and across different pathologies.

## Caption Electronic Supplementary Material

Survey of the study

## Abbreviations

ICU: Intensive care unit
FICUS: Family intensive care unit syndrome
EC: Ethics committee
