# Supplementary material for: Prioritizing information topics for relatives of critically ill patients: Cross-sectional survey among intensive care unit relatives and professionals
Source: Wien Klin Wochenschr. 2018 Aug 9;130(21):645–52. doi: 10.1007/s00508-018-1377-1 (PMC6244832; doi:10.1007/s00508-018-1377-1)
Supplement: Supplementary file 1 — Survey of the study [file 508_2018_1377_MOESM1_ESM.pdf]

Sehr geehrte Angehörige!

Ihre Meinung und Ihr Wohlbefinden sind uns wichtig!

Daher wollen wir in unserer wissenschaftlichen Erhebung um Ihre Meinung bitten, um die Kommunikation und die Information der Angehörigen/Familien auf der Intensivstation zu verbessern.

Ihre Teilnahme an dieser Befragung erfolgt freiwillig und dauert ca. 7- 9 Minuten. Bitte machen Sie auch gerne von der offenen Frage am Ende der Befragung gebrauch, welche einen freien Texteintrag zulässt. Wir freuen uns über Feedback und Anregungen.

Bitte geben Sie den ausgefüllten Fragebogen in das unbeschriftete Kuvert und verschließen Sie dieses, bevor Sie es bei Ihrer Station abgeben. Die Kuverts werden nur von den ForschungsleiterInnen geöffnet.

Die Ergebnisse werden gesammelt und es werden dann ausführliche Informationen, welche genauer auf die Bedürfnisse von Angehörigen/Familien abgestimmt sind, zur Verfügung gestellt.

Mit freundlichen Grüßen

Mag. Magdalena Hoffmann, MSc, MBA
Assoz. Prof. Dr. Karin Amrein. MSc

Haben Sie noch Fragen?

Mag. in Magdalena Hoffmann, MSc, MBA
Kontaktdaten der Forscherin

Bitte kreuzen Sie an: Angaben zur Person:

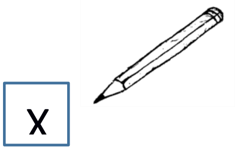

1. Bitte wählen Sie Ihr Geschlecht aus!

Weiblich	☐	Männlich	☐

2. Wie alt sind Sie? (Bitte Alter in Jahren angeben z.B. 46)

3. In welcher Beziehung stehen Sie zum/zur Patient/in?

Ehefrau/Ehemann	☐	Schwester/Bruder	☐
Partner/Partnerin	☐	Enge Freunde	☐
Eltern	☐	Tante/Onkel	☐
Sohn/Tochter	☐	Sonstiges	☐

4. Bevor Ihr Angehöriger auf die Intensivstation eingeliefert wurde, hatten Sie schon einmal Erfahrung als Angehörige/r eines/er Intensivpatienten/in?

Ja	☐	Nein	☐

5. Leben Sie mit dem /der Patient/in einem Haushalt zusammen?

Ja	☐	Nein	☐

6. Ihre höchste abgeschlossene Schulbildung/Ausbildung?

Hauptschule	☐	Meister	☐
Lehre	☐	Fachschule	☐
HTL	☐	Matura	☐
Kolleg	☐	FH	☐
Universität	☐	Sonstiges	☐

Über welche medizinischen Themen wünschen Sie sich mehr vertrauenswürdige und gut verständliche Information? Bitte markieren Sie nach Ihrer Priorität!

**7. DIAGNOSE (Feststellung der Krankheit)**	Nicht wichtig	Weniger wichtig	Mittelmäßig wichtig	Ziemlich wichtig	Sehr wichtig	Nicht relevant
Neurologischer Status (z.B. das Bewusstsein, sehen können)	☐	☐	☐	☐	☐	☐
Fieber (z.B. Risiko und Folgen, Unterkühlung)	☐	☐	☐	☐	☐	☐
Krankheiten (z.B. häufige Erkrankungen und Komplikationen)	☐	☐	☐	☐	☐	☐
Erscheinungsbild (z.B. starke Rötung oder Blässe)	☐	☐	☐	☐	☐	☐
Vitalfunktionen (z.B. Blutdruck, Atemfrequenz, Herzschlag)	☐	☐	☐	☐	☐	☐
Untersuchungen (z.B. Röntgen, Herzultraschall)	☐	☐	☐	☐	☐	☐

**8. BEHANDLUNG UND THERAPIE**						
Operationen(z.B. Herzoperation, Magenoperation)	☐	☐	☐	☐	☐	☐
Behandlung & Therapie (z.B. neueste Therapiemethoden)	☐	☐	☐	☐	☐	☐
Entwöhnung vom Beatmungsgerät (z.B. Dauer, Komplikationen)	☐	☐	☐	☐	☐	☐
Beatmungsgerät (z.B. Schläuche-u. Gerätefunktionen)	☐	☐	☐	☐	☐	☐
Medikamente (z.B. Wirkung, Nebenwirkungen)	☐	☐	☐	☐	☐	☐

**9. PROGNOSE (Vorhersage über den Verlauf)**						
Krankheitsdauer (z.B. Zeit bis zur Gesundung)	☐	☐	☐	☐	☐	☐
Todesfall und Trauer (z.B. Mit wem kann ich sprechen, was ist zu tun?)	☐	☐	☐	☐	☐	☐
Wahrscheinlichkeiten/Annahmen (z.B. Wie geht es nach der Intensivstation weiter?)	☐	☐	☐	☐	☐	☐
Auskunftsrechte und Information (z.B. Warum brauchen wir ein Codewort? Wer darf Informationen erhalten?)	☐	☐	☐	☐	☐	☐
Krisen (z.B. akute Verschlechterung der Werte oder der Psyche)	☐	☐	☐	☐	☐	☐

10. KOMFORT (Rund ums Wohlbefinden)

	Nicht wichtig	Weniger wichtig	Mittelmäßig wichtig	Ziemlich wichtig	Sehr wichtig	Nicht relevant
Psychische Belastung (z.B. Angst, Stress bei PatientInnen und Angehörigen)	☐	☐	☐	☐	☐	☐
Wohlbefinden steigern (z.B. für PatientInnen und Angehörige)	☐	☐	☐	☐	☐	☐
Körperliche Schmerzen (z.B. Hat mein Angehöriger Schmerzen? Was wird gegen die Schmerzen getan?)	☐	☐	☐	☐	☐	☐
Ernährung (z.B. durch die Magensonde, durch eine Infusion)	☐	☐	☐	☐	☐	☐
Schlafen (z.B. Warum schläft mein Angehöriger?)	☐	☐	☐	☐	☐	☐

11. INTERAKTION (Kommunikation mit Ihrem Angehörigen auf der Intensivstation)

Sprechen (z.B. Auf welchen Wegen kann mein Angehöriger mit mir sprechen?)	☐	☐	☐	☐	☐	☐
Antworten (z.B. Kann mein Angehöriger mir antworten?)	☐	☐	☐	☐	☐	☐
Berührungen (z.B. Darf ich meinen Angehörigen berühren?)	☐	☐	☐	☐	☐	☐
Hören (z.B. Kann mein Angehöriger mich hören?)	☐	☐	☐	☐	☐	☐
Meine Mithilfe (z.B. Was kann ich beitragen?)	☐	☐	☐	☐	☐	☐

12. KOMMUNIKATION (Informationsaustausch mit der Intensivstation)

Termine auf der Intensivstation (z.B. Tagesablauf, wann kann ich meinen Angehörigen besuchen, wann kann ich mit einem Arzt sprechen?)	☐	☐	☐	☐	☐	☐
Information erhalten (z.B. Folder, Internet)	☐	☐	☐	☐	☐	☐
News (z.B. Neuigkeiten über die Intensivstation)	☐	☐	☐	☐	☐	☐
Team (z.B. Wer hat welche Aufgabe bei der Behandlung und Betreuung?)	☐	☐	☐	☐	☐	☐
Telefon (z.B. Wo kann ich wann anrufen?)	☐	☐	☐	☐	☐	☐

13. FAMILIE/ANGEHÖRIGE

	Nicht wichtig	Weniger wichtig	Mittelmäßig wichtig	Ziemlich wichtig	Sehr wichtig	Nicht relevant
Besuchszeiten (z.B. Wer darf wann kommen?)	☐	☐	☐	☐	☐	☐
Keime im Krankenhaus (z.B. Was ist für mich als Angehöriger wichtig zu wissen z.B. Händedesinfektion, Isolierung)	☐	☐	☐	☐	☐	☐
Familienkonferenz (z.B. Entscheidungsfindung)	☐	☐	☐	☐	☐	☐
Belastung und Sorge (z.B. Wo gibt es Hilfe?)	☐	☐	☐	☐	☐	☐
Religion (z.B. Wie kann ich einen religiösen Beistand für uns finden?)	☐	☐	☐	☐	☐	☐

14. NACH DER INTENSIVSTATION

Länge des Aufenthalts (z.B. Durchschnittliche Aufenthaltsdauer)	☐	☐	☐	☐	☐	☐
Rückfall (z.B. Was kann vorsorglich getan werden?)	☐	☐	☐	☐	☐	☐
Folgeerkrankungen (z.B. Mangelernährung, Wundliegen, Immobilität)	☐	☐	☐	☐	☐	☐
Verlegung (z.B., wo kommt mein Angehöriger hin und warum? Tipps für die Entlassung)	☐	☐	☐	☐	☐	☐
Erinnerungen (z.B. Tagebuch auf der Station)	☐	☐	☐	☐	☐	☐

15. TOD UND TRAUER

Tod und Trauer (z.B. Abschiednehmen, Kindern den Tod erklären)	☐	☐	☐	☐	☐	☐

16. INTERNET - Benutzen Sie das Internet um sich über Gesundheitsthemen zu informieren?

Nie	☐
Selten	☐
Manchmal	☐
Oft	☐
Immer	☐

17. Haben Sie bereits im Internet nach Informationen rund um den Gesundheitszustand und den Intensivstationsaufenthalt Ihres Angehörigen gesucht?

Ja ☐ Nein ☐

18. Haben Sie noch Fragen? Wurde ein wichtiges Thema vergessen? Möchten Sie uns etwas mitteilen?

……………………………………………………………………………………………………

……………………………………………………………………………………………………

……………………………………………………………………………………………………

………………………………………………………………………………

Herzlichen Dank für Ihre Unterstützung!

Abgabe: Die Abgabe der Fragebögen erfolgt anonym. Bitte geben Sie den ausgefüllten Fragebogen in das unbeschriftete Kuvert und verschließen Sie dieses. Danach geben Sie es bitte auf Ihrer Intensivstation ab. Die Kuverts werden ungeöffnet den ForscherInnen übergeben.
